# Quantitative analysis of contrast-enhanced ultrasound in neoadjuvant treatment of locally advanced rectal cancer: a retrospective study

**DOI:** 10.3389/fonc.2023.1340060

**Published:** 2024-01-23

**Authors:** Gouyang Bai, Congying Wang, Yi Sun, Jinghua Li, Xiangzhou Shi, Wei Zhang, Yilin Yang, Ruijing Yang

**Affiliations:** ^1^ Department of Ultrasound, Tang Du Hospital, Xi’an, China; ^2^ Department of Clinical Laboratory, Tang Du Hospital, Xi’an, China; ^3^ Department of Pathology, Tang Du Hospital, Xi’an, China

**Keywords:** ultrasonography, neoadjuvant therapy, locally advanced rectal cancer, prognosis, colorectal cancer

## Abstract

**Purpose:**

To explore the clinical value of contrast-enhanced ultrasound (CEUS) quantitative analysis in the evaluation and prognosis of neoadjuvant chemoradiotherapy for locally advanced rectal cancer (LARC).

**Methods:**

Eighty-three consecutive patients undergoing neoadjuvant chemoradiotherapy and total mesorectal excision for LARC were retrospectively included. According to pathological results, patients were categorized into complete or incomplete response groups. Differences in ultrasonic parameters, pathological results, and clinical data between groups were evaluated. The cutoff point for a complete response as determined by quantitative analysis of CEUS was assessed using a receiver operating characteristic curve; additionally, overall survival (OS) and progression-free survival (PFS) were analyzed.

**Results:**

Of the 83 patients, 12 (14.5%) achieved a complete response and 71 (85.5%) did not. There were significant between-group differences in carcinoembryonic antigen (CEA) levels, differentiation degree, proportion of tumor occupying the lumen, anterior-posterior and superior-inferior diameters of the lesion, and intensity of enhancement (P<0.05). CEUS quantitative analysis showed significant between-group differences in peak intensity (PI) and area under the curve (AUC) values (P<0.05). The OS and PFS of patients with high PI, high AUC value, and poorly differentiated cancer were significantly worse than those with low PI, low AUC values, and moderately to highly differentiated cancer (P<0.05). High CEA levels (hazard ratio: 1.02, 95% confidence interval: 1.01–1.04; P=0.002) and low differentiation (2.72, 1.12–6.62; P=0.028) were independent risk factors for PFS and OS.

**Conclusions:**

CEUS can predict the response to neoadjuvant treatment in patients with LARC. CEUS quantitative analysis is helpful for clinical prognosis.

## Introduction

Colorectal cancer ranks high in both morbidity and mortality rates worldwide (1, 2). In recent years, total mesorectal excision (TME) has become the standard treatment for rectal cancer, while neoadjuvant chemoradiotherapy (NCRT) has substantially improved the control of local lesions in patients with locally advanced rectal cancer (LARC). This has led to increased survival rates for patients with rectal cancer (3, 4). Therefore, the efficacy of neoadjuvant treatment and its impact on patient prognosis have garnered much clinical attention. Timely observation of the efficacy of neoadjuvant treatment is of great importance for selecting appropriate clinical treatment measures; some strictly selected patients can even achieve a complete response after NCRT with a “wait and see” policy and avoid surgical treatment (5, 6).

In patients with LARC, the use of magnetic resonance imaging (MRI)-based radiomics has demonstrated a certain effect in predicting a complete response as well as survival outcomes after chemoradiotherapy (7, 8). Transrectal ultrasound has been utilized in staging rectal cancer and in assessing responses to neoadjuvant treatment. Several studies have reported that sequential endorectal ultrasonography examinations can predict the effectiveness of preoperative chemoradiotherapy as a treatment for LARC (9–11). Contrast-enhanced ultrasound (CEUS) is a widely accepted and extensively used imaging modality that can quantitatively evaluate tumor microvascular blood-flow perfusion information (12, 13). However, the role of CEUS-derived blood-flow information in assessing the efficacy of chemoradiotherapy and predicting survival outcomes in patients with LARC has not yet been reported.

Thus, this study aimed to investigate the relationship between post-neoadjuvant treatment CEUS parameters of LARC and the pathological results and clinical data of these patients to evaluate the effect of quantitative parameters on the prognosis of patients after TME surgery.

## Materials and methods

### Study population

We retrospectively reviewed 83 consecutive patients diagnosed with rectal cancer based on pathology at our hospital between May 2017 and December 2021. We collected the patients’ ultrasound parameters, pathological results, and clinical data and conducted follow-ups to ascertain survival outcomes. Moreover, all ultrasound parameter data were collected after neoadjuvant treatment and 1 week prior to surgery. Inclusion criteria were a clinical diagnosis of LARC with neoadjuvant treatment prior to surgery, a distance of 12cm from the lower margin of the tumor to the anal margin, and the availability of complete and analyzable CEUS data of the lesion. Exclusion criteria were failure to complete the planned neoadjuvant treatment or radical surgery, the simultaneous presence of multiple primary malignant tumors, or loss to follow-up.

The protocol of this study was approved by the Institutional Review Board of our hospital Clinical Research Ethics Committee (protocol number: 201901–03; date of approval: January 29, 2019). The trial was registered at Chinese Clinical Trials.gov: No. ChiCTR1900022298.

### Treatment methods

All enrolled patients received NCRT, with a total radiation dosage of 50–55 Gy delivered in 1.8–2.0 Gy fractions over 25–28 sessions, and concomitant chemotherapy consisting of 5-fluorouracil or capecitabine. Radical surgery treatment was performed at 6–8 weeks after completion of neoadjuvant treatment.

### Follow-up definitions

Progression-free survival (PFS) was defined as the time/duration from the initial diagnosis at our hospital until local recurrence, distant metastasis, or death prior to surgery. Overall survival (OS) was defined as the duration from the initial diagnosis to death or the end of the follow-up period. All patients underwent routine clinical examinations every 3 months during the first-year post-surgery and every 6 months thereafter. Each examination included a review of the clinical data, serum testing, and chest-abdomen-pelvis computed tomography (CT).

### Instrument and methods

A LOGIQ E9 ultrasound scanner (GE Healthcare, Chicago, IL), equipped with low mechanical index ultrasound imaging technology, was used with a transrectal endoscopic probe with a frequency of 5–9 MHz and SonoVue contrast agent (Bracco, Milan, Italy). The patient was given an enema 1 h prior to the examination and then placed in a left lateral position with the hip and knee in flexion. The location of the lesion was determined under two-dimensional ultrasound, and the thickness, cumulative length, and percentage of the intestinal lumen occupied by the tumor were recorded by repeated multi-section scanning. Subsequently, when the blood flow was most abundant in the lesion and some normal intestinal wall was displayed simultaneously, the CEUS mode was switched on, and 2.4 mL of ultrasound contrast agent microbubble suspension was injected into the cubital vein cluster, followed by rapid injection of 5 mL of saline to allow CEUS examination of the primary tumor lesion. Using the dual-phase contrast interface, the enhancement of the contrast agent was observed in real-time, and images were continuously stored and recorded for 90 s each. The instrument’s integral measurement software was used to obtain values for contrast-related parameters, and the rectal tumor was selected as the region of interest (ROI). The ROI, where the contrast agent was uniformly and steadily distributed, was manually adjusted, and the mucosal layer of the normal intestinal wall at 1 cm away from the tumor was selected as the control area. The software automatically draws the time-intensity curve of the contrast agent perfusion in the ROI, including its rise time (RT), time to peak enhancement (TTP), peak intensity (PI), ascending slope (AS), and area under the curve (AUC). The time-intensity curve was measured continuously five times, and the average values were obtained. The enhancement mode was divided into high enhancement, iso-enhancement, and low enhancement, based on the contrast between the lesion and the normal mucosal layer of the rectal wall. Image analysis was performed in a blinded fashion by two ultrasound physicians with over 10 years of experience.

### Statistical analysis

SPSS version 26.0 (IBM, Armonk, NY) was employed for statistical analyses. According to the pathological results, the patients were divided into complete and incomplete pathological response groups. Categorical variables were compared between groups using the chi-squared test. For differences in measures between groups, we used the *t*-test when the data conformed to a normal distribution. We used the Mann–Whitney U test for non-normally distributed parameters and plotted the receiver operating characteristic (ROC) curve to evaluate the diagnostic efficacy. Kaplan–Meier survival and Cox regression analyses were performed based on pathological results, clinical data, and CEUS parameters. A p-value of less than 0.05 was considered to be statistically significant.

## Results

### Comparison of baseline characteristics and CEUS methods

The 83 patients with LARC who underwent neoadjuvant treatment had adenocarcinoma confirmed by postoperative pathology and included 32 (37.3%) cases of poorly differentiated carcinoma and 51 (57.8%) cases of moderately to highly differentiated carcinoma. Moreover, 25 (30.1%) cases were lymph node-positive and 58 (69.9%) were lymph node-negative. Twelve (14.5%) patients achieved a complete response, while 71 (85.5%) patients had a partial response. Complete response to neoadjuvant treatment for rectal cancer was related to carcinoembryonic antigen (CEA) levels and tumor differentiation, with significant differences (P=0.041 and 0.045, respectively). However, it was not associated with sex or age. Complete response to neoadjuvant treatment for rectal cancer was significantly associated with the proportion of the bowel lumen occupied by the tumor, the anterior-posterior and superior-inferior diameters of the lesion, and the intensity of enhancement (all p<0.05) (**Table 1**).

**Table 1:** Comparison of baseline characteristics and CEUS methods.

Characteristic		Complete response (n=12)	Incomplete response (n=71)	*x* ^2^/Z	P-value
Sex					
	Male	4 (33.3)	45 (63.4)	2.690	0.101
	Female	8 (66.7)	26 (36.6)		
Age, years					
	<60	6 (50.0)	39 (54.9)	0.100	0.751
	≥60	6 (50.0)	32 (45.1)		
CEA (ug/L)					
	<5	12 (100)	47 (66.2)	4.180	0.041
	≥5	0 (0)	24 (33.8)		
Differentiation					
	Poorly differentiated	2 (16.7)	30 (42.3)	4.020	0.045
	Moderately to highly differentiated	10 (83.3)	41 (57.7)		
Extent of tumor infiltration					
	<1/2	12 (100)	47 (66.2)	4.18	0.041
	≥1/2	0 (0)	24 (33.8)		
Intensity of enhancement					
	Low enhancement	8 (66.7)	21 (29.6)	4.687	0.030
	High enhancement	4 (33.3)	50 (70.4)		
**Anterior-posterior diameter (cm)** ^*^		0.60 ± 0.32	1.11 ± 0.50	-3.685	<0.001
**Superior-inferior diameter (cm)** ^*^		1.75 ± 0.98	2.60 ± 1.50	-3.980	<0.001

Data are n (%) unless otherwise indicated.

^*^Data are medians and interquartile range, and Mann–Whitney U test was used.

CEUS, contrast-enhanced ultrasound; CEA, carcinoembryonic antigen.

### Quantitative analysis of CEUS images in neoadjuvant treatment for rectal cancer

**Figure 1:**
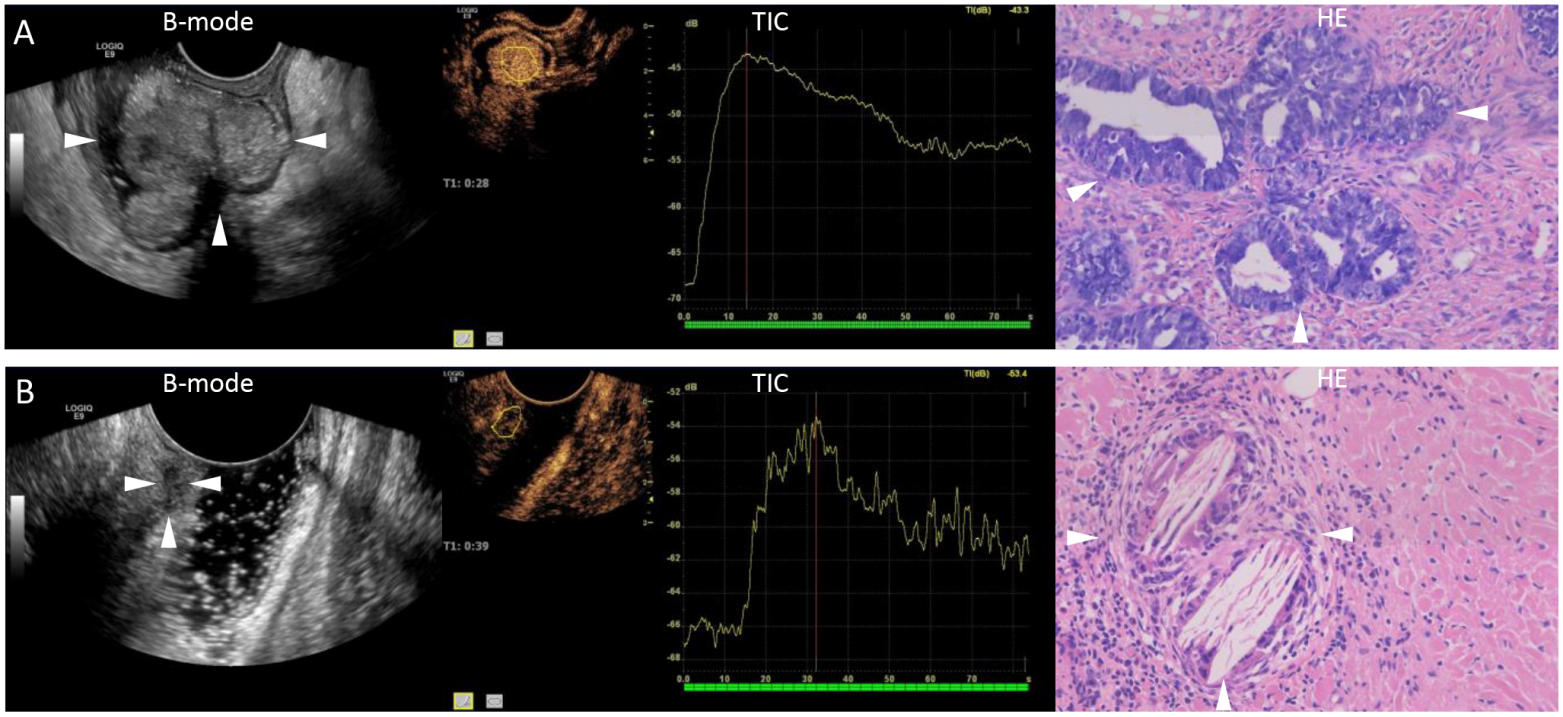
Representative images of ultrasound and pathology after neoadjuvant chemoradiotherapy. **(A)** A 76-year-old male patient with a B-mode ultrasound showing the extent of rectal lesions (arrows). Quantitative analysis of contrast-enhanced ultrasound shows high enhancement, with a peak intensity of 24.9 dB and an area under the curve of 1065.70 dB. Pathology showing incomplete remission (arrows, hematoxylin stain, original magnification ×20). **(B)** A 52-year-old female patient with a B-mode ultrasound showing the extent of rectal lesions (arrows). Quantitative analysis of contrast-enhanced ultrasound shows low enhancement, with a peak intensity of 13.6 dB and an area under the curve of 524.58 dB. Pathology showing complete remission (arrows, hematoxylin stain, original magnification ×20). B-mode, brightness-mode; TIC, time-intensity curve; HE, hematoxylin stain.

The results of the CEUS quantitative analysis indicated that the AUC and PI values in the group with an incomplete pathological response following neoadjuvant treatment for rectal cancer were significantly higher than those in the group with a complete response (both P<0.05). However, no significant differences were observed in RT, TTP, or AS between the two groups (all P>0.05) (**Table 2**; **Figure 1**).

**Table 2:** Quantitative analysis of CEUS in NCRT for rectal cancer.

Parameter	RT^*^	TTP^#^	AS^*^	AUC^#^	PI^*^
**Complete response** **(n=12)**	9.00 ± 3.50	27.17 ± 8.05	2.39 ± 1.20	829.63 ± 131.91	21.25 ± 4.22
**Incomplete response** **(n=71)**	9.00 ± 6.00	27.04 ± 7.19	2.28 ± 1.42	966.00 ± 117.22	24.50 ± 3.40
***t*/Z**	-0.560	0.003	-0.123	13.408	-3.737
**P-value**	0.576	0.957	0.902	0.001	<0.001

^*^Data are medians and interquartile range, and Mann–Whitney U test was used.

^#^Data are mean ± standard deviation, and Student’s t-test was used.

CEUS, contrast-enhanced ultrasound; NCRT, neoadjuvant chemoradiotherapy, RT, rise time; TTP, time to peak enhancement; AS, ascending slope; AUC, area under the curve; PI, peak intensity.

### PI and AUC evaluation for complete response after neoadjuvant treatment for rectal cancer

**Figure 2:**
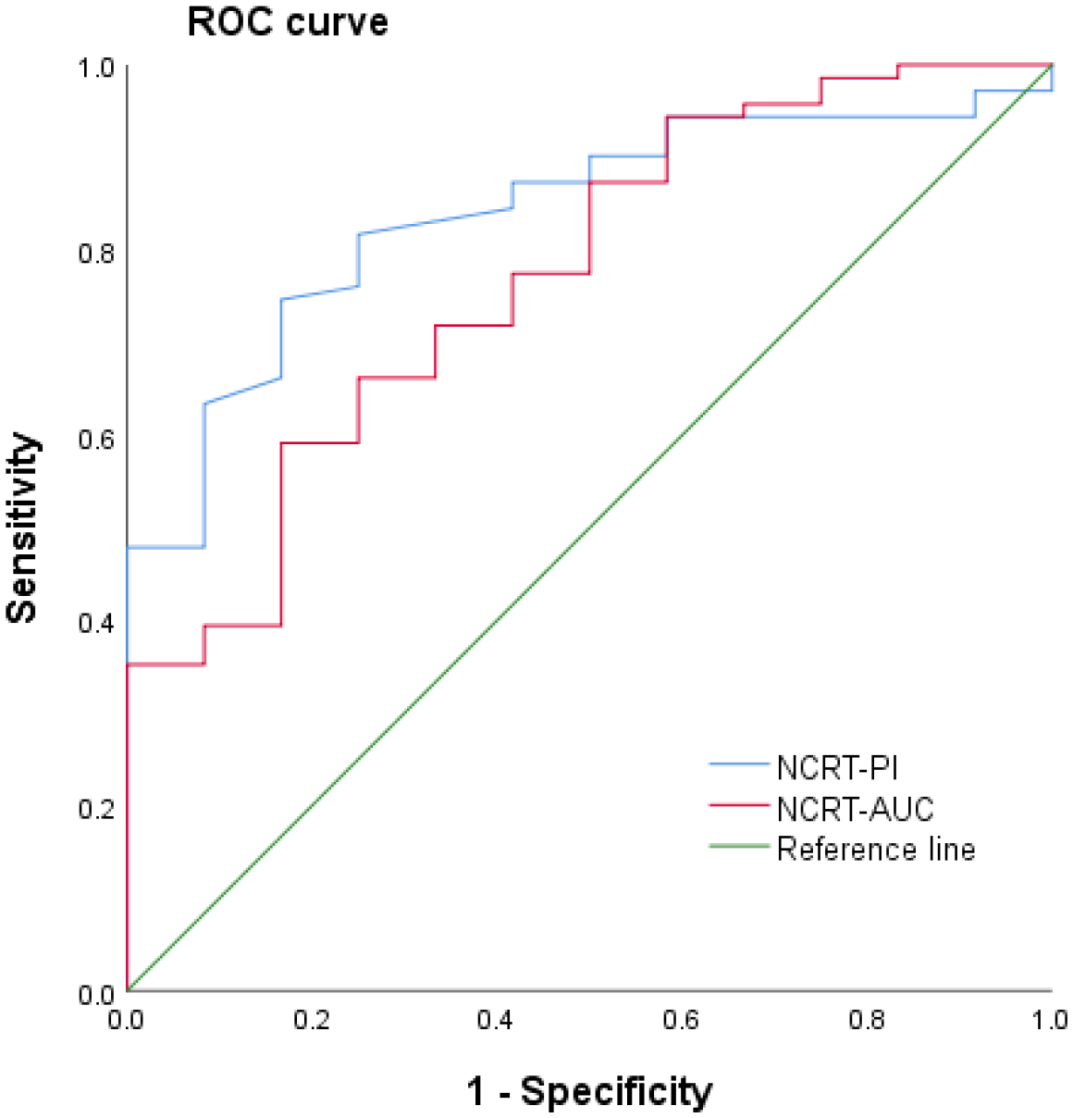
Receiver operating characteristic (ROC) curve of contrast-enhanced ultrasound quantitative analysis for evaluating a complete response following neoadjuvant treatment for rectal cancer. NCRT, neoadjuvant chemoradiotherapy; AUC, area under the curve; PI, peak intensity.

Using ROC analysis, cutoff values of 23.1 dB and 938.56 dB were selected for the PI and AUC, respectively, to evaluate the sensitivity and specificity of a complete response after neoadjuvant treatment for rectal cancer. The sensitivity and specificity were 76.4% and 83.3% for PI, and 64.6% and 83.3% for AUC (**Figure 2**).

### Prognostic analysis

**Figure 3:**
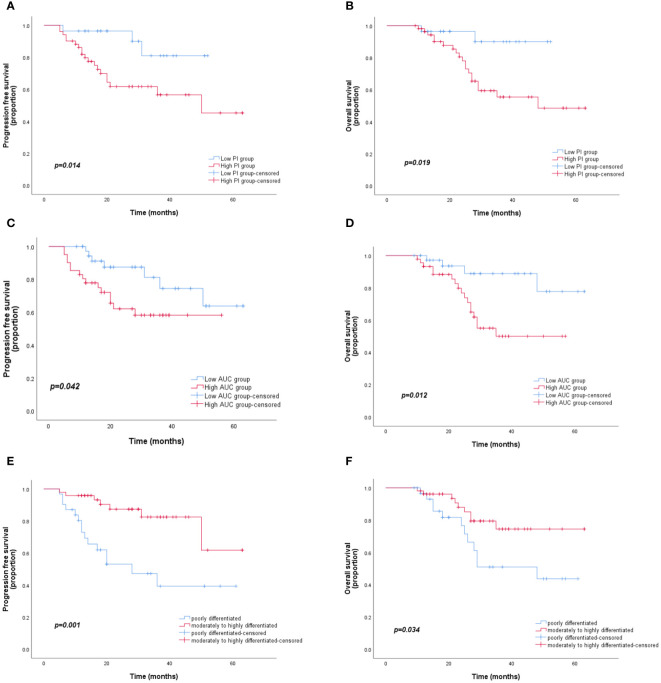
Kaplan–Meier curves for PFS and OS according to the AUC, PI, and differentiation degree. **(A, B)** OS and PFS for different PI groups. **(C, D)** OS and PFS for different AUC groups. **(E, F)** OS and PFS for different degrees of differentiation. PFS, progression-free survival; OS, overall survival; AUC, area under the curve; PI, peak intensity.

Among the 83 patients with LARC, the median follow-up time was 27 months, with a maximum follow-up time of 63 months. At the last follow-up, 19 patients died, 3 had *in situ* recurrence, and 21 had distant metastasis, mainly to the liver, lungs, and pelvic lymph nodes. Based on the PI grouping by ROC curve, Kaplan–Meier analysis showed that the PFS and OS of the low-PI group were significantly better than those of the high-PI group (P=0.014 and 0.019, respectively). Moreover, significant differences were observed in PFS and OS between the low- and high-AUC groups (P=0.042 and 0.012, respectively). Furthermore, the PFS and OS of the moderately to highly differentiated group were significantly superior to those of the poorly differentiated group (P=0.001 and 0.034, respectively) (**Figure 3**). In the Cox regression analysis, the univariate analysis results indicated that CEA, differentiation degree, lymph node metastasis, AUC, and PI were key predictors of PFS and OS. The multivariate analysis revealed that high CEA levels (hazard ratio [HR]: 1.02, 95% confidence interval [CI]: 1.01–1.04; P=0.002) and low differentiation (HR: 2.72, 95% CI: 1.12–6.62; P=0.028) were independent risk factors for PFS and OS (**Table 3**).

**Table 3:** Univariate and multivariate analyses of prognostic factors for PFS and OS.

Analysis	PFS		OS	
	HR (95% CI)	P-value	HR (95% CI)	P-value
Univariable				
**CEA (ug/L)**	1.03 (1.01, 1.05)	<0.001	1.02 (1.01, 1.03)	0.004
**Differentiation (poorly vs. moderately to highly)**	0.19 (0.07, 0.48)	<0.001	0.34 (0.14, 0.82)	0.017
**Lymph node metastasis (no vs. yes)**	3.85 (1.64, 9.07)	0.002	3.43 (1.44, 8.16)	0.005
**Anterior-posterior diameter (cm)**	0.88 (0.51, 1.51)	0.649	0.98 (0.66, 1.48)	0.939
**Superior-inferior** **diameter (cm)**	1.01 (0.77, 1.33)	0.944	1.04 (0.79, 1.38)	0.758
**AUC**	1.01 (1.00, 1.01)	0.017	1.01 (1.00, 1.01)	0.011
**PI**	1.22 (1.03, 1.43)	0.018	1.21 (1.02, 1.44)	0.031
Multivariable				
**CEA (ug/L)**	1.03 (1.01, 1.05)	0.001	1.02 (1.01, 1.04)	0.011
**Differentiation (moderately to highly vs. poorly)**	5.15 (1.98, 13.39)	0.003	2.70 (1.12, 6.62)	0.031
**AUC**	1.00 (1.00, 1.01)	0.062	1.00 (1.00, 1.01)	0.045

PFS, progression-free survival; OS, overall survival; HR, hazard ratio; CI, confidence interval; CEA, carcinoembryonic antigen; AUC, area under the curve; PI, peak intensity.

## Discussion

For the treatment of LARC, current guidelines recommend a comprehensive strategy of preoperative NCRT and radical TME. Previous studies have reported that 15–20% of patients with rectal cancer who undergo neoadjuvant treatment achieve a pathological complete response (pCR) (14). In the present study, the pCR rate reached 14.5%, which was consistent with the values reported in the literature, indicating that NCRT has a significant therapeutic effect on progressive rectal cancer and can help change or determine the subsequent treatment strategy to some extent. The degree of tumor response to NCRT is an indicator of clinical efficacy. Therefore, accurately evaluating the therapeutic effect of neoadjuvant treatment, particularly in patients with pCR, is a challenging topic in clinical research.

Currently, the clinical imaging methods that have been used to evaluate the efficacy of neoadjuvant treatment for rectal cancer primarily include CT, MRI, and positron emission tomography-CT, with MRI with different sequences being the primary choice. Studies have confirmed that both MRI functional parameters, such as apparent diffusion coefficient values and vascular perfusion parameters, are reliable predictors of prognosis in patients with rectal cancer (15–17). However, rectal MRI requires special coil preparation, particular postures, and involves a cumbersome operation, as well as discomfort to the patient due to the confined space, which limits the use of MRI to examine rectal cancer. The literature has revealed that transrectal ultrasound is highly accurate in the preoperative staging of rectal cancer (18). However, due to swelling, inflammation, fibrosis, and necrosis of the rectal tumor and the surrounding structures induced by radiotherapy and chemotherapy, transrectal ultrasound cannot accurately identify the tumor margin. Hence, it is difficult to assess the efficacy of neoadjuvant treatment for rectal cancer accurately by ultrasound. Antitumor therapies, such as radiotherapy and chemotherapy, can alter the hemodynamic parameters related to blood-flow perfusion within the tumor (19). Ultrasound contrast agent microbubbles are pure blood-pool contrast agents that always flow in the blood circulation after intravenous injection and do not penetrate outside blood vessels, making them an ideal tracer for studying tissue blood perfusion (20, 21). In this study, ultrasound contrast and quantitative analysis were used to evaluate the efficacy of neoadjuvant treatment for rectal cancer. Our results showed that the proportion of the tumor occupying the intestinal lumen, the anterior-posterior and superior-inferior diameters of the lesion, and the enhancement intensity of the ultrasound contrast after neoadjuvant treatment were smaller in the complete response group than in the incomplete response group, indicating that the treatment effect was better. The tumor shrinkage was more obvious in the complete response group. The quantitative analysis results revealed that the PI and AUC values of rectal cancer lesions after neoadjuvant treatment were significantly lower in the complete response group than in the incomplete response group. This indicated that the pathological microscopic changes after radiotherapy mainly involved neovascularization and necrosis of tumor cells, while CEUS could reflect changes in hemodynamic parameters related to blood-flow perfusion in tumor tissues, regardless of the enhancement mode or quantitative analysis. ROC curve analysis showed that the sensitivity of PI and AUC values in evaluating complete response following neoadjuvant treatment for LARC was 76.4% and 64.6%, respectively, while the specificity was 83.3% for both (**Figure 2**). Accordingly, the use of CEUS and quantitative measurement of PI and AUC values, in addition to routine ultrasound examination, demonstrated high sensitivity and specificity for distinguishing a complete response after neoadjuvant treatment for LARC, which can serve as a reference for clinical judgment of the efficacy of neoadjuvant treatment.

The prognosis of LARC treated with neoadjuvant treatment and TME is of great concern to clinicians and patients. Early evaluation and analysis of complete response or partial regression of tumors following neoadjuvant treatment play a vital role in improving the long-term prognosis of patients with LARC. The literature shows that CEA, the degree of differentiation, and the extent of tumor infiltration are independent risk factors for rectal cancer (22, 23), and that CEA and the degree of differentiation are also poor prognostic factors for combined neoadjuvant treatment and TME in LARC (24–26). Our results were essentially consistent with these findings (**Table 3**; **Figure 3**). The Kaplan–Meier analysis demonstrated that the PFS and OS of patients with rectal cancer with low PI and AUC values who underwent neoadjuvant treatment combined with TME were significantly better than those of rectal cancer patients with high PI and AUC values.

This study has several limitations. First, our study only discussed the difference between patients with complete and incomplete responses. In patients with incomplete response, there are individuals who have a good or poor response to neoadjuvant therapy, but we have not conducted further studies. At the same time, compared with the number of patients with incomplete response, the number of patients with complete response was smaller and there was a quantitative imbalance between the two. In order to reduce allocation bias, we will again subdivide and classify patients with incomplete response in future studies. Secondly, because ultrasound is affected by human factors, we used the same sonographer who has been in the field for more than 10 years to collect images, and asked for measurements at the same level, and took five measurements to calculate the average.

In conclusion, our results indicate that quantitative analysis of CEUS can be used to evaluate the efficacy of neoadjuvant treatment in patients with progressive rectal cancer and that this may become a new reference index for assessing the degree of relief and changes in the effectiveness of neoadjuvant treatment in clinical practice. In future studies, more sensitive CEUS parameters should be explored, and the sample size should be increased to verify the utility of CEUS quantitative parameters in the clinical evaluation of the effectiveness of neoadjuvant treatment for rectal cancer.

## Data availability statement

The raw data supporting the conclusions of this article will be made available by the authors, without undue reservation.

## Ethics statement

The studies involving humans were approved by Institutional Review Board of Tangdu Hospital, Fourth Military Medical University Clinical Research Ethics Committee (protocol number: 201901–03; date of approval: January 29, 2019). The studies were conducted in accordance with the local legislation and institutional requirements. The participants provided their written informed consent to participate in this study.

## Author contributions

GB: Conceptualization, Writing – original draft. CW: Data curation, Formal Analysis, Writing – original draft. YS: Data curation, Writing – review & editing, Resources. JL: Data curation, Writing – review & editing, Resources. XS: Data curation, Resources, Writing – review & editing. WZ: Data curation, Resources, Writing – review & editing. RY: Writing – review & editing, Conceptualization, Project administration, Supervision, Writing – original draft. YY: Project administration, Supervision, Writing – original draft, Writing – review & editing, Methodology, Validation.
